# Three-Dimensional Growth of Prostate Cancer Cells Exposed to Simulated Microgravity

**DOI:** 10.3389/fcell.2022.841017

**Published:** 2022-02-17

**Authors:** Dorothea Dietrichs, Daniela Grimm, Jayashree Sahana, Daniela Melnik, Thomas J. Corydon, Markus Wehland, Marcus Krüger, Randy Vermeesen, Bjorn Baselet, Sarah Baatout, Trine Engelbrecht Hybel, Stefan Kahlert, Herbert Schulz, Manfred Infanger, Sascha Kopp

**Affiliations:** ^1^ Department of Microgravity and Translational Regenerative Medicine, Otto von Guericke University Magdeburg, Magdeburg, Germany; ^2^ Research Group “Magdeburger Arbeitsgemeinschaft für Forschung unter Raumfahrt- und Schwerelosigkeitsbedingungen” (MARS), Otto von Guericke University Magdeburg, Magdeburg, Germany; ^3^ Department of Biomedicine, Aarhus University, Aarhus, Denmark; ^4^ Department of Ophthalmology, Aarhus University Hospital, Aarhus, Denmark; ^5^ Radiobiology Unit, SCK CEN, Belgian Nuclear Research Centre, Mol, Belgium; ^6^ Department of Molecular Biotechnology, Ghent University, Ghent, Belgium; ^7^ Institute of Anatomy, Otto von Guericke University Magdeburg, Magdeburg, Germany

**Keywords:** prostate cancer, cytokines, interleukins, cytoskeleton, extracellular matrix, PAM signaling, prostate cancer cells and microgravity

## Abstract

Prostate cancer metastasis has an enormous impact on the mortality of cancer patients. Factors involved in cancer progression and metastasis are known to be key players in microgravity (µ*g*)-driven three-dimensional (3D) cancer spheroid formation. We investigated PC-3 prostate cancer cells for 30 min, 2, 4 and 24 h on the random positioning machine (RPM), a device simulating µ*g* on Earth. After a 24 h RPM-exposure, the cells could be divided into two groups: one grew as 3D multicellular spheroids (MCS), the other one as adherent monolayer (AD). No signs of apoptosis were visible. Among others, we focused on cytokines involved in the events of metastasis and MCS formation. After 24 h of exposure, in the MCS group we measured an increase in *ACTB, MSN, COL1A1, LAMA3, FN1, TIMP1, FLT1, EGFR1, IL1A, IL6, CXCL8*, and *HIF1A* mRNA expression, and in the AD group an elevation of *LAMA3, COL1A1, FN1*, *MMP9*, *VEGFA, IL6,* and *CXCL8* mRNAs compared to samples subjected to 1 *g* conditions. Significant downregulations in AD cells were detected in the mRNA levels of *TUBB, KRT8*, *IL1B, IL7, PIK3CB, AKT1 and MTOR* after 24 h. The release of collagen-1α1 and fibronectin protein in the supernatant was decreased, whereas the secretion of IL-6 was elevated in 24 h RPM samples. The secretion of IL-1α, IL-1β, IL-7, IL-2, IL-8, IL-17, TNF-α, laminin, MMP-2, TIMP-1, osteopontin and EGF was not significantly altered after 24 h compared to 1 *g* conditions. The release of soluble factors was significantly reduced after 2 h (IL-1α, IL-2, IL-7, IL-8, IL-17, TNF-α, collagen-1α1, MMP-2, osteopontin) and elevated after 4 h (IL-1β, IL-2, IL-6, IL-7, IL-8, TNF-α, laminin) in RPM samples. Taken together, simulated µ*g* induced 3D growth of PC-3 cancer cells combined with a differential expression of the cytokines IL-1α, IL-1β, IL-6 and IL-8, supporting their involvement in growth and progression of prostate cancer cells.

## Introduction

As estimated by GLOBOCAN (Global Cancer Observatory) in 2020, prostate cancer (PC) comprises an incidence of almost 1.4 million new cases and 375,000 deaths worldwide (Sung et al., 2021). PC was the second most frequent cancer and the fifth leading cause of cancer death among men in 2020 (Sung et al., 2021). Adenocarcinomas are the most common types of PC, and in general PC progresses very slowly. The 5-years survival rate for most men with local or regional PC is nearly 100%, but if diagnosed with PC metastasis, the 5-years survival rate is reduced to 31% (Gandaglia et al., 2014).

This shows that metastasis of PC has a vast impact on the mortality and the overall quality of life of patients. Compared to other cancer types, PC metastasizes predominantly to the skeleton (84%) and the lymph nodes (10.6%) (Gandaglia et al., 2014). Additionally, spreading to the liver (10.2%) and thorax (9.1%) is also common (Gandaglia et al., 2014). Metastatic PC is mostly terminal even after intensive multimodal treatment. Therefore, there is an urgent need to increase the knowledge of PC biology, genomics, proteomics and advanced profiling technologies in order to find new drug development targets.

In context of PC expansion, proteins released by the PC cells (PCC) into the interstitial space are of high interest. Secreted factors such as cytokines and chemokines are released into the tumor microenvironment and play a key role in cancer progression. Cytokines are released in response to immune reactions like infection, inflammation and immunity in order to inhibit tumor development and progression. The tumor cells can respond just as well to cytokines that induce cancer growth, reduce programmed cell death and facilitate invasion and metastasis (Dranoff, 2004). Thus, cytokines, their receptors and specific signaling pathways are key factors in driving the specific events leading to metastasis of PC (Dranoff, 2004; Adekoya and Richardson, 2020).

Moreover, cytokines are key players in all events of the metastatic process and hence, they remodel the extracellular matrix, influence the epithelial-mesenchymal-transition, invasion, angiogenesis, and the processes involved in the establishment of tumor cells in the secondary organs (Adekoya and Richardson, 2020).

An extraordinary and novel approach to investigate tumor cell processes is using microgravity (μ*g*), either with real (r-) μ*g* in space or simulated (s-) μ*g* by ground-based devices like the random positioning machine (RPM) (Becker and Souza, 2013). Space provides special physical conditions which cannot be reproduced on Earth, as well as μ*g* conditions which are used to investigate molecular mechanisms and signaling processes controlling cell growth and function (Becker and Souza, 2013). Cancer research in space and molecular biological studies on cells exposed to r- and/or s-μ*g* are therefore a hot topic in space medicine (Krüger et al., 2019; Grimm et al., 2020; Nassef et al., 2020).

Previous research revealed that r- and s-μ*g* have a large impact on the biochemistry and physiology of human cells. This comprises various changes, such as alterations of the extracellular matrix (ECM), the focal adhesion complex, the cytoskeleton, growth behavior, as well as changes in differentiation and proliferation (Nassef et al., 2019). Moreover, Häder *et al.* (Häder et al., 2017) suggested a direct correlation of the µ*g*-induced cytoskeletal changes and transcriptional alterations. They concluded that the interaction of the ECM, cell adhesion and the cytoskeleton is of great importance for gravisensing in human cells. Cytoskeletal alterations detected in human cells exposed to µ*g* were described as follows: microtubules are regularly localized more around the nucleus and might lose their radial organization, are shortened, as well as more curved and bent. The F-actin network is altered and the number of stress fibers reduced. F-actin is redistributed and is situated more perinuclear or is localized more cortical. Intermediate filaments (vimentin, cytokeratin) form clusters, reveal larger meshes and are localized more perinuclear (Vorselen et al., 2014). These findings obtained from fixed cells were recently confirmed by live-cell imaging in r-µ*g* (Corydon et al., 2016a; Nassef et al., 2019).

Moreover, s- and r-µ*g* influenced ECM proteins in a time-dependent manner (Infanger et al., 2006) and cell-type dependent, resulting in increases or decreases of ECM components (Infanger et al., 2006; Marrero et al., 2009; Zhivodernikov et al., 2020). In addition, PC MCS engineered on the RPM revealed a decrease in *COL1A1* after 3 days and an increase after 5 days, whereas basement components like *COL4A5* and *LAMA3* as well as the cell adhesion molecule *FN1* were elevated in MCS at both time points (Hybel et al., 2020). These findings indicate that the cells try to expand the ECM in MCS to stabilize themselves and to resist the s-μ*g* conditions, as the ECM provides structural support for the cells (Bonnans et al., 2014).

In addition, lack of gravity and/or RPM exposure of various cell types was shown to promote cell growth in a scaffold-free three-dimensional (3D) way, forming MCS (Riwaldt et al., 2016; Grimm et al., 2020). MCS are 3D aggregates exhibiting complex cell-to-cell and cell-to-matrix interactions. These interactions have been reported to induce gradients for nutrients, gases, growth factors and signal factors. This 3D structure reflects the natural microenvironment of cells more accurately than 2D monocultures and also resembles the microenvironment of real tissues (Mehta et al., 2012; Cui et al., 2017). Several cancer cell-types like thyroid and breast cancer cells exposed to an RPM formed MCS within 24 h (Kopp et al., 2016; Riwaldt et al., 2016).

3D PC aggregates (PC-3, LNCaP and DU-145 cell lines) engineered on microgravity simulators and the subsequent formation of 3D spheroids was demonstrated on both, the NASA rotary cell culture system and the RPM (Ingram et al., 1997; Hybel et al., 2020).

Understanding the biology of spheroids is very important for a more complete appreciation of *in vivo* tissue formation and function. MCS are frequently used to study molecular mechanisms involved in angiogenesis, cancer development, and biology and for pharmacological testing. Unveiling the mechanisms of microgravity-dependent molecular and cellular changes is an up-to-date requirement for improving space medicine and cancer research (Becker and Souza, 2013; Krüger et al., 2019; Nassef et al., 2020). A clear advantage of microgravity is that it enables the engineering of MCS without any scaffolds. Moreover, long-term experiments using thyroid cancer cells show that FTC-133 spheroids and EA.hy926 spheroids or intima constructs did not develop a central necrosis, when exposed to an RPM (Kopp et al., 2015; Dittrich et al., 2018).

Therefore, spheroids formation in µ*g* is an innovative approach to study the early phases of tumor progression and metastasis. The PI3K/AKT/mTOR (PAM) signaling pathway is frequently mutated in prostate cancer and thus a good candidate for the involvement in tumor progression (Tee et al., 2018). It is regulating growth, metabolism, and migration of PCC.

In this study we investigated the impact of short-term (30 min, 2, 4 and 24 h) s-µ*g*-exposure via RPM on PC-3 prostate cancer cells, which were established from an adenocarcinoma. The principal aim of this study was to measure the gene expression and secretion rate of cytokines in PCC. Secondly, we focused on the altered gene expression of cytoskeletal factors and the extracellular matrix (ECM). Third, a special focus was placed on the PAM signaling pathway, which is proposed to be the underlying mechanism of spheroid formation in PC. Fourth, we engineered 3D spheroids under 1*g-*conditions by the liquid-overlay technique to use them as a control for the 3D spheroids in simulated microgravity and studied the gene expression of selected factors in 1*g-*MCS.

## Materials and Methods

### Cell Cultures

The PC-3 cell line (ECACC 90112714) was purchased from the European Collection of Authenticated Cell Cultures (ECACC). The cells originated from a 62-year-old male Caucasian suffering from grade 4 prostatic adenocarcinoma.

3·10^6^ cells were seeded into T75 cm^2^ flasks (Sarstedt, Nümbrecht, Germany) and cultured using RPMI 1640 medium (Gibco, Fisher Scientific, Schwerte, Germany), supplemented with 10% FCS (Sigma Aldrich, Steinheim, Germany) and 1% penicillin/streptomycin (Life Technologies, New York, United States). Every 3 days the medium was changed, and upon reaching 70–80% confluence, the cells were split at a 1:10 ratio.

### Simulated Microgravity on the iRPM and Sample Collection

In preparation for the experiments in s-µ*g* on the incubator RPM (iRPM), 10 T25 cm^2^ flasks (Sarstedt, Nümbrecht, Germany; order nr. 83.3910.002 vented caps) per group were filled each with 2·10^6^ cells in 13 ml RPMI 1640 medium (Life Technologies, Paisley, United Kingdom), complemented with 10% FCS (Sigma Aldrich, Steinheim, Germany) and 1% penicillin/streptomycin (Life Technologies, New York, United States), and kept in 1 *g* conditions (37°C, 5% CO_2_) for 1 day to let the cells adhere. Afterwards, the flasks were filled entirely with growth medium, avoiding the formation of air bubbles. Furthermore, the bottle caps were secured at the edges with parafilm, sparing the ventilated area. Five flasks of each group were placed and fixed inside the incubator on the iRPM, while the other five remained under 1*g* standard conditions (both 37°C, 5% CO_2_). After the duration of 30 min, 2 h, and 4 h, respectively, the cell culture supernatants were collected in 50 ml tubes and stored at −150°C. Then 2 ml RNA*later* Stabilization Solution (Invitrogen by Thermo Fischer Scientific, Waltham, MA, United States) was added to the flasks and the cells mechanically detached with cell scrapers. The resulting cell suspensions were collected in 15-ml tubes and stored at 4°C until further processing.

For the immunofluorescence staining, 0.2·10^5^ cells were seeded into 4 slideflasks (Thermo Scientific) and incubated for 36 h. At this timepoint the culture medium was discarded, the slideflasks completely filled with fresh RPMI 1640 medium (Life Technologies, Paisley, United Kingdom) containing 10% FCS (Sigma Aldrich, Steinheim, Germany) and 1% penicillin/streptomycin (Life Technologies, New York, United States), and sealed with parafilm air bubble-free. Following, two flasks were cultured on the iRPM for 24 h, while two were left in the incubator under standard conditions as controls. After the experiment, the medium was discarded and the slides were fixed with 4% Paraformaldehyde (PFA, Sigma-Aldrich, St. Louis, Missouri, United States) in phosphate-buffered saline (PBS; Gibco, Life Technologies, Paisley, United Kingdom).

The iRPM was constructed by the group of Professor Jörg Sekler at the Fachhochschule Nordwestschweiz (Windisch AG, Switzerland). Details on the iRPM are described in (Benavides Damm et al., 2014).

### Liquid Overlay

The liquid-overlay technique is an established method to generate 3D cell aggregates in static culture (Svejgaard et al., 2015). In short, 96-well plates are coated with 40 µl of 1% agarose in RPMI 1640 medium. After hardening of the gel, 4,000 cells/200 µl in RPMI 1640 supplemented with 10% FCS and 1% Pen/Strep were incubated as described in 4.1 for 24 h. This step was followed by microscopic evaluation of cell viability using Ready Probes for live cell imaging (Thermo Scientific, Waltham, Massachusetts, United States) and cell aggregate collection for quantitative real-time PCR. Five 96-well plates were seeded and spheroids of one plate were collected to make up one PCR sample. As a control, adherently growing cells were cultured for 24 h in five standard T25 cm^2^ cell culture flasks.

### Quantitative Real-Time Polymerase Chain Reaction (qPCR)

The expression levels of the genes of interest were determined via qPCR. Primer Express software (Applied Biosystems) was used to design appropriate primers with a T_m_ of ∼60°C (Table 1). The primers were synthesized by TIB Molbiol (Berlin, Germany) and all assays were run on a 7,500 Fast Real-Time PCR System using the FAST SYBR™ Green Master Mix (both Applied Biosystems, Darmstadt, Germany). The reaction volume was 15 μL including 1 μL of template cDNA and a final primer concentration of 500 nM. PCR conditions were as follows: 20 s at 95°C, 40 cycles of 30 s at 95°C and 30 s at 60°C, followed by a melting curve analysis step (temperature gradient from 60 to 95°C with +0.3°C/cycle).

**TABLE 1:** Primers used for qPCR analyses.

Gene	Primer name	Sequence 5′–3′
*18S rRNA*	18s-F	GGAGCCTGCGGCTTAATTT
	18s-R	CAACTAAGAACGGCCATGCA
*ACTB*	ACTB-F	TGCCGACAGGATGCAGAAG
	ACTB-R	GCCGATCCACACGGAGTACT
*AKT1*	AKT1-F	CTTCTATGGCGCTGAGATTGTG
	AKT1	CAGCATGAGGTTCTCCAGCTT
*CASP3*	CASP3-F	CTCCAACATCGACTGTGAGAAGTT
	CASP3-R	GCGCCAGCTCCAGCAA
*CASP8*	CASP8-F	TGCAAAAGCACGGGAGAAAG
	CASP8-R	CTCTTCAAAGGTCGTGGTCAAAG
*CASP9*	CASP9-F	CTCCAACATCGACTGTGAGAAGTT
	CASP9-R	GCGCCAGCTCCAGCAA
*COL1A1*	COL1A1-F	ACGAAGACATCCCACCAATCAC
	COL1A1-R	CGTTGTCGCAGACGCAGAT
*CXCL8*	CXCL8-F	TGGCAGCCTTCCTGATTTCT
	CXCL8-R	GGGTGGAAAGGTTTGGAGTATG
*EGF*	EGF-F	TGCCAGCTGCACAAATACAGA
	EGF-R	TCTTACGGAATAGTGGTGGTCATC
*EGFR*	EGFR-F	TTGCCGCAAAGTGTGTAACG
	EGFR-R	GAGATCGCCACTGATGGAGG
*EZR*	EZR-F	GCAATCCAGCCAAATACAACTG
	EZR-R	CCACATAGTGGAGGCCAAAGTAC
*FLT1*	FLT1-F	CCCTCGCCGGAAGTTGTAT
	FLT1-R	GATAATTAACGAGTAGCCACGAGTCAA
*FN1*	FN1-F	AGATCTACCTGTACACCTTGAATGACA
	FN1-R	CATGATACCAGCAAGGAATTGG
*HIF1A*	HIF1A-F	TGCTTTAACTTTGCTGGCCC
	HIF1A-R	AGTTTCTGTGTCGTTGCTGC
*IL1A*	IL1A-F	AGTAGCAACCAACGGGAAGG
	IL1A-R	AGGCTTGATGATTTCTTCCTCTGA
*IL1B*	IL1B-F	TTCGAGGCACAAGGCACAA
	IL1B-R	TGGCTGCTTCAGACACTTGAG
*IL6*	IL6-F	CGGGAACGAAAGAGAAGCTCTA
	IL6-R	GAGCAGCCCCAGGGAGAA
*IL7*	IL7-F	CCAGTTGCGGTCATCATGACTA
	IL7-R	TGATGCTACTGGCAACAGAACA
*KDR*	KDR-F	TCTTCTGGCTACTTCTTGTCATCATC
	KDR-R	GATGGACAAGTAGCCTGTCTTCAGT
*KRT8*	KRT8-F	GATCTCTGAGTGAACCGGAACA
	KRT8-R	GCTCGGCATCTGCAATGG
*LAMA3*	LAMA3-F	AAAGCAAGAAGTCAGTCCAGC
	LAMA3-R	TCCCATGAAGACCATCTCGG
*MMP9*	MMP9-F	CCTGGAGACCTGAGAACCAATC
	MMP9-R	TTCGACTCTCCACGCATCTCT
*MSN*	MSN-F	GAAATTTGTCATCAAGCCCATTG
	MSN-R	CCATGCACAAGGCCAAGAT
*MTOR*	MTOR-F	ATCTTGGCCATAGCTAGCCTC
	MTOR-R	ACAACTGGGTCATTGGAGGG
*PIK3CB*	PIK3CB-F	AGAAAAGTTTGGCCGGTTCC
	PIK3CB-R	GCAGTCAACATCAGCGCAAA
*RDX*	RDX-F	GAAAATGCCGAAACCAATCAA
	RDX-R	GTATTGGGCTGAATGGCAAATT
*SPP1*	SPP1-F	CGAGGTGATAGTGTGGTTTATGGA
	SPP1-R	CGTCTGTAGCATCAGGGTACTG
*TGFB1*	TGFB1-F	CACCCGCGTGCTAATGGT
	TGFB1-R	AGAGCAACACGGGTTCAGGTA
*TIMP1*	TIMP1-F	GCCATCGCCGCAGATC
	TIMP1-R	GCTATCAGCCACAGCAACAACA
*TUBB*	TUBB-F	CTGGACCGCATCTCTGTGTACTAC
	TUBB-R	GACCTGAGCGAACAGAGTCCAT
*VEGFA*	VEGFA-F	CTACCTCCACCATGCCAAGTG
	VEGFA-R	GCGCTGATAGACATCCATGAAC

If all amplicons showed one single T_m_ similar to the one predicted by the Primer Express software, the PCR reactions were considered specific. Every sample was measured in triplicates, and relative quantification was performed by means of the comparative C_T_ (ΔΔC_T_) method. *18S rRNA* was used as a housekeeping gene to normalize the expression data.

### Immunofluorescence

Following the removal of PFA, the cells were washed twice with PBS and permeabilized with 0.2% Triton. After blocking with 3% bovine serum albumin (BSA) in PBS for 1 h, the primary antibody was added and incubated at 4°C overnight. The next day, the cells were washed twice with PBS, the secondary antibody was added and incubated for 1 h. Afterwards, the cells were rinsed three times with PBS and mounted with DAPI fluoroshield and a cover slip. A list of antibodies and probes used for immunofluorescence staining is given in Table 2.

**TABLE 2:** Materials used for immunofluorescence.

Antibody/Probe	Species	Order number	Manufacturer	Dilution in PBS
Fibronectin	mouse monoclonal	sc-18827	Santa Cruz Biotechnology	(1:100)
anti-mouse IgG (H + L)	goat secondary antibody	A11001	Invitrogen by Thermo Fischer Scientific	(1:500)

### Multiplex Bead Array

Collagen I alpha I, fibronectin, interleukin (IL)-1α/1F1, IL-1β/1F2, IL-2, IL-6, IL-7, IL-8/CXCL8, IL-17/17-A, tumor necrosis factor alpha (TNF-α), matrix metalloproteinase-2 (MMP-2), epidermal growth factor (EGF), serpin E1/plasminogen activator inhibitor-1 (PAI-1), osteopontin (OPN), chemokine (C-C motif) ligand 2 (CCL2)/monocyte chemoattractant protein 1 (MCP-1), tissue inhibitor metalloproteinases metallopeptidase inhibitor 1 (TIMP-1) and laminin levels in cell culture supernatant were analyzed using a multiplex magnetic bead array (R&D systems, Minneapolis, United States). Assays were performed according to manufacturer’s instructions. Samples were run on a MAGPIX instrument (Luminex, s-Hertogenbosch, Netherlands) and analyzed with MILLIPEX analyst standard version 5.1 (Merck, Darmstadt, Germany).

### Microscopy

After immunofluorescence staining, the slides were investigated using confocal laser scanning microscopy. The observations were made with a Leica DM 2000 microscope equipped with a 40x objective and an external light source Leica EL 6000 (Leica Microsystems GmbH, Wetzlar, Germany).

### Statistical Analysis

The statistics were performed using the GraphPad Prism 7.01 software (GraphPad Software, Inc., California, United States). Differences between s-µ*g* samples and related controls were assessed with the Mann-Whitney U-test, *p*-values < 0.05 were considered significant.

## Results

### Cell Growth, Morphology and Cell Viability

**FIGURE 1:**
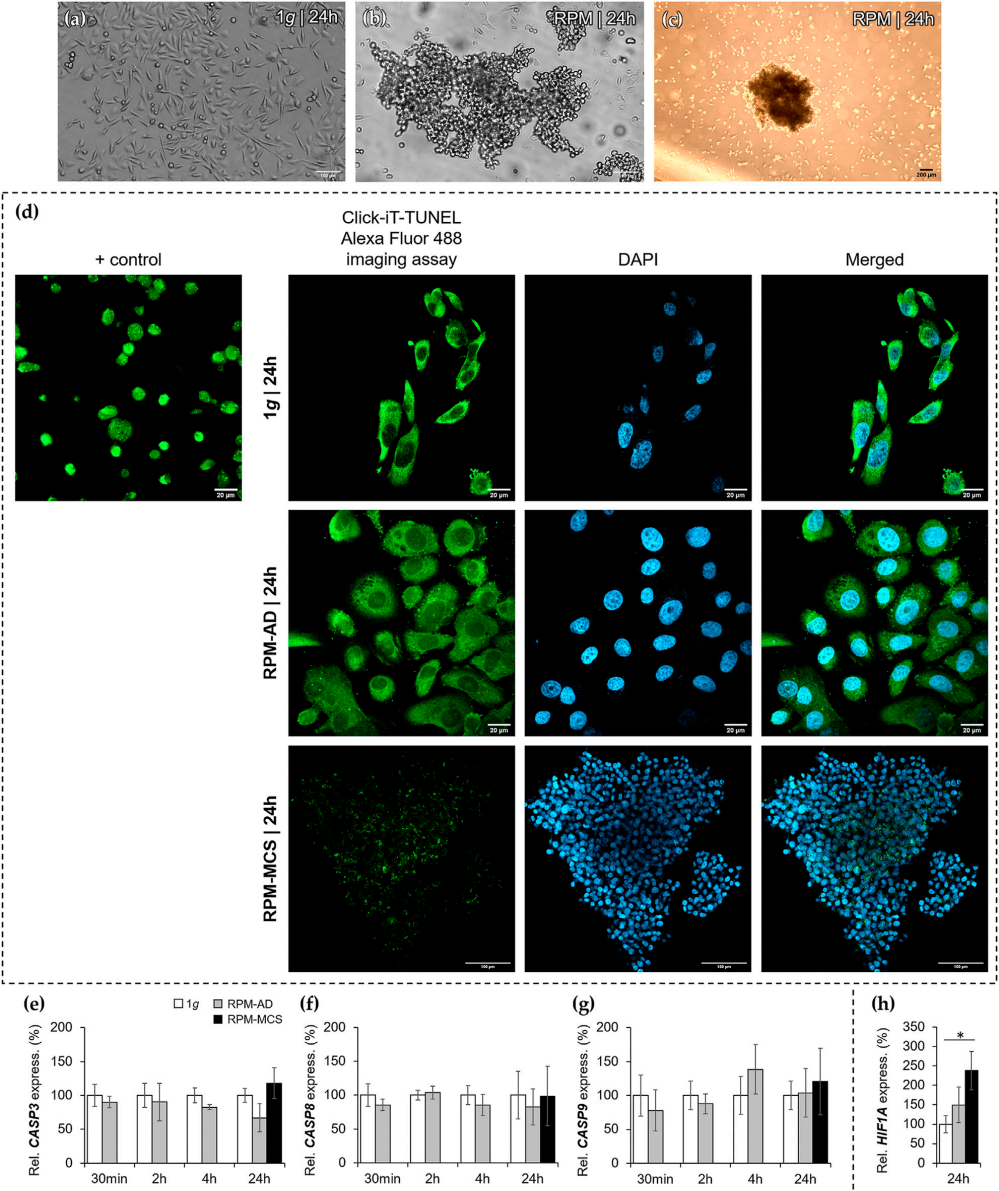
Phase contrast microscopy of PC-3 cells: **(A)** static 1*g* control cells (Scale bar 100 µm) and **(B,C)** 24 h RPM-exposed samples showing detached spheroids swimming above adherently growing cells (scale bar **(B)** 100 µm and **(C)** 200 µm). **(D)** Terminal deoxynucleotidyl transferase dUTP nick end labeling (TUNEL) assay and 4′,6-diamidino-2-phenylindole (DAPI) staining revealed no apoptotic cells in 1*g* controls (scale bar 20 µm) and also no apoptosis in RPM-exposed AD (scale bar 20 µm and MCS cells (scale bar 100 µm). The positive control, induced by DNase, is given in the first row (Scale bar 20 µm). **(E,F,G)** The *CASP3, CASP8* and *CASP9* (30 min, 2, 4 and 24 h) gene expression was not significantly altered at all time points. **(H)** The gene expression of *HIF1A* after 24 h was not changed in AD cells, but significantly elevated in MCS compared to 1*g* (n = 5).

Culture flasks containing 70% sub-confluent PC-3 cells were mounted on the RPM for 30 min, 2, 4 and 24 h. The 1*g* control samples were placed next to the RPM and cultured in parallel. PC-3 cells cultured under 1*g*-conditions grew as 2D monolayer cultures (Figure 1A). PC-3 cells exposed to the RPM for 30 min, 2 and 4 h showed no three-dimensional (3D) growth and grew adherently on the cell culture flask bottom (not shown). Phase contrast microscopy showed normal epithelial PC-3 cells exhibiting numerous microvilli, abnormal nuclei and nucleoli. The cells subjected to short-term exposure (30 min, 2 h, and 4 h) to the RPM compared to 1*g* samples revealed no visible morphological changes. No dead cells were detectable. 3D multicellular spheroids could be detected in the supernatant after a 24 h RPM exposure. Thus, there are two different phenotypes of PC-3 cells visible: adherently growing cells (AD) and detached 3D MCS (Figures 1B,C) We used a “terminal deoxynucleotidyl transferase dUTP nick end labeling” (TUNEL) assay to detect DNA breaks formed during the final phase of apoptosis, when DNA fragmentation takes place. No apoptotic cells were visible in all AD cell samples irrespective of RPM exposure or not. Sporadic apoptotic cells were detected in MCS (Figure 1D). In addition, we focused on apoptosis signaling. Genes associated with apoptosis such as *CASP3*, *CASP8,* and *CASP9* mRNAs were not significantly changed after short-term incubation (30 min, 2 h, and 4 h) as well as after 24 h on the RPM compared to 1*g* (Figures 1E–G). We studied the gene expression of *HIF1A* (hypoxia inducible factor 1) in PC-3 cells exposed for 24 h to s-µ*g* conditions. The *HIF1A* mRNA in AD cells was not altered, but significantly elevated in MCS (Figure 1H).

### Spheroids Engineered Under 1*g*-Conditions

**FIGURE 2:**
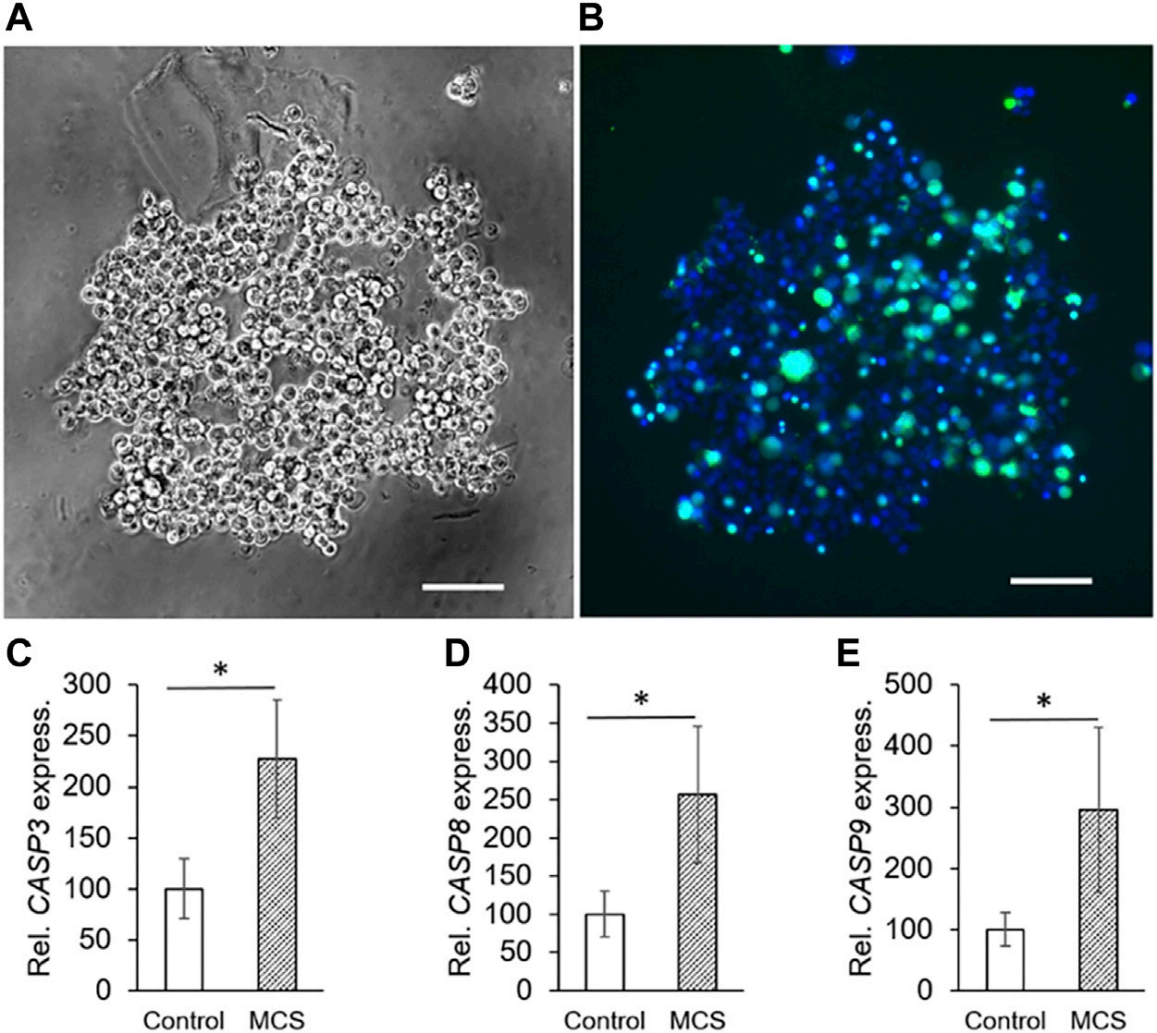
Phase contrast microscopy of PC-3 cells: **(A)** static 1*g-*MCS after 24 h using the liquid overlay technique (scale bar 100 µm) and **(B)** same sample using the live cell viability assay, where the blue color indicates the nuclei of all cells while the green color demonstrates compromised cells. **(C,D,E)** The *CASP3, CASP8* and *CASP9* (24 h) gene expression was significantly altered in 1*g*-MCS after 24 h (n = 5).

The liquid-overlay technique was used to obtain MCS under 1*g*-conditions within 24 h. We microscopically investigated the MCS formation and their viability. Figure 2A presents a representative cell aggregate formed within 24 h. In comparison to the MCS built on the RPM, the cells are loosely united. Figure 2B presents the cell viability staining. While all nuclei are stained blue, compromised nuclei, representing non-viable cells, are stained green. Compared to the MCS engineered on the RPM, the cell viability of MCS formed under 1*g*-conditions (Figure 2B) seems to be lower. The gene expression of *CASP3*, *CASP8* and *CASP9* (Figures 2C–E) was significantly upregulated in 1*g*-MCS compared to control samples.

### The Cytoskeleton

**FIGURE 3:**
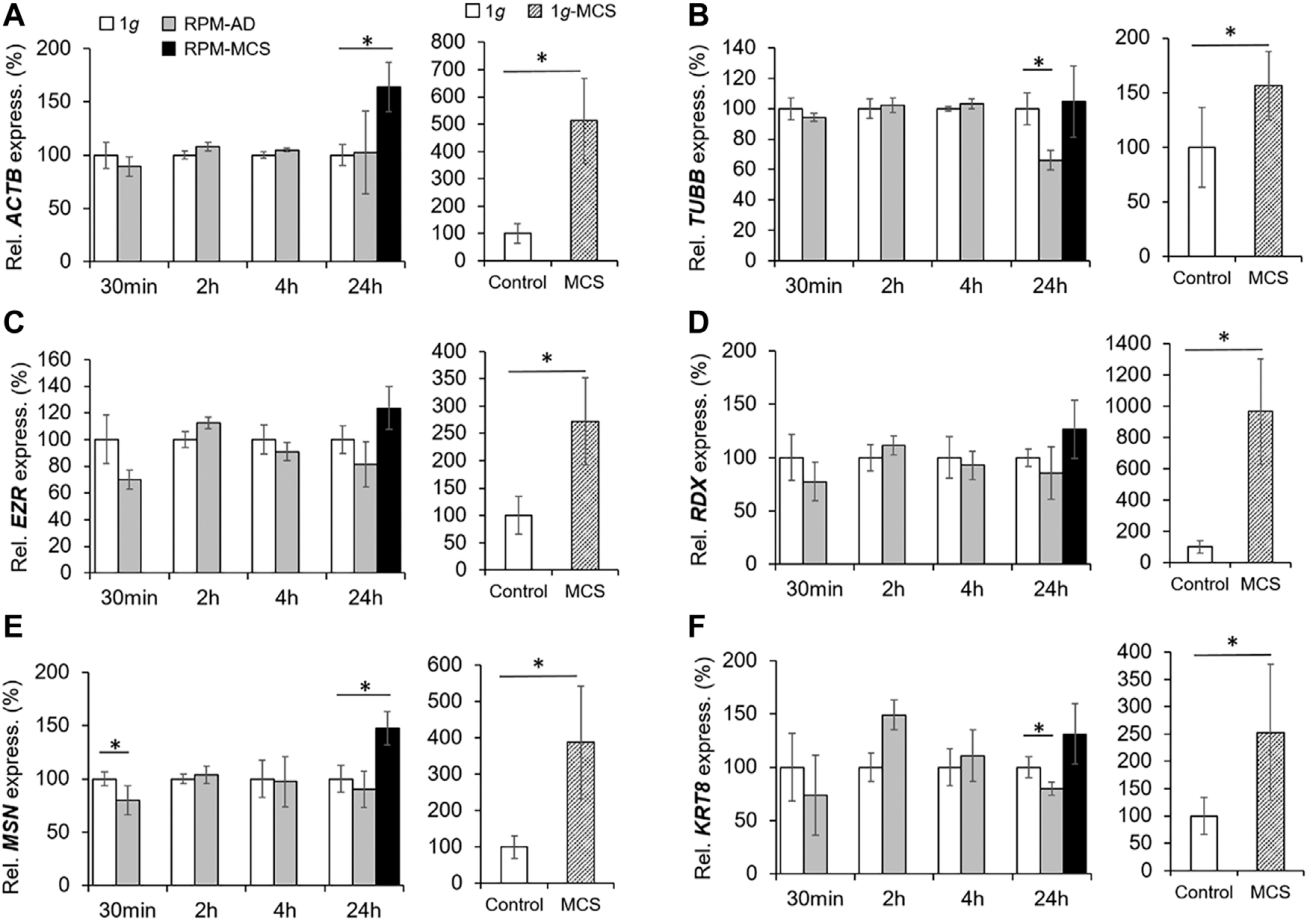
Gene expression of **(A)** *ACTB,* **(B)** *TUBB,* **(C)** *EZR,* **(D)** *RDX,* **(E)** *MSN* and **(F)** *KRT8* of PC-3 cells exposed to the RPM for 30 min, 2, 4 and 24 h and 1*g*-MCS after 24 h. n = 5; *p < 0.05 vs. 1*g*.

The cytoskeletal protein β-actin (ACTB) is widely distributed in all eukaryotic cells and is involved in cell migration, cell division, cell structure, cell integrity and immune response. After 24 h RPM exposure an increase in the *ACTB* gene expression was measured in MCS, but not in the adherent cells compared to 1*g* (Figure 3A). MCS built under 1*g*-conditions showed a significant upregulation of *ACTB* in comparison to control samples. A short-term (30 min, 2 h, and 4 h) RPM exposure of PC-3 cells did not change the gene expression level of *ACTB*.

The *TUBB* gene expression did not change when PC-3 cells were exposed to short-term s-µ*g* (Figure 3B). In contrast, AD cells exhibited a downregulated *TUBB* expression after 24 h compared to 1*g* cells. PC-3 cells growing in MCS did not show a change in *TUBB* expression. No significant differential *EZR* and *RDX* gene expression could be observed over the experiment duration (Figure 3C, Figure 3D respectively). Interestingly, the MSN mRNA was downregulated after a 30-min RPM exposure, whereas the gene was upregulated in MCS after 24 h compared to corresponding static 1*g* samples (Figure 3E). In addition, the *KRT8* mRNA was significantly downregulated in AD after 24 h compared to 1*g* (Figure 3F). While in RPM samples were only marginal expression changes visible, the investigations of these genes in 1*g*-MCS showed a significant upregulation of the cytoskeletal genes (Figures 3A–F).

### The Extracellular Matrix

**FIGURE 4:**
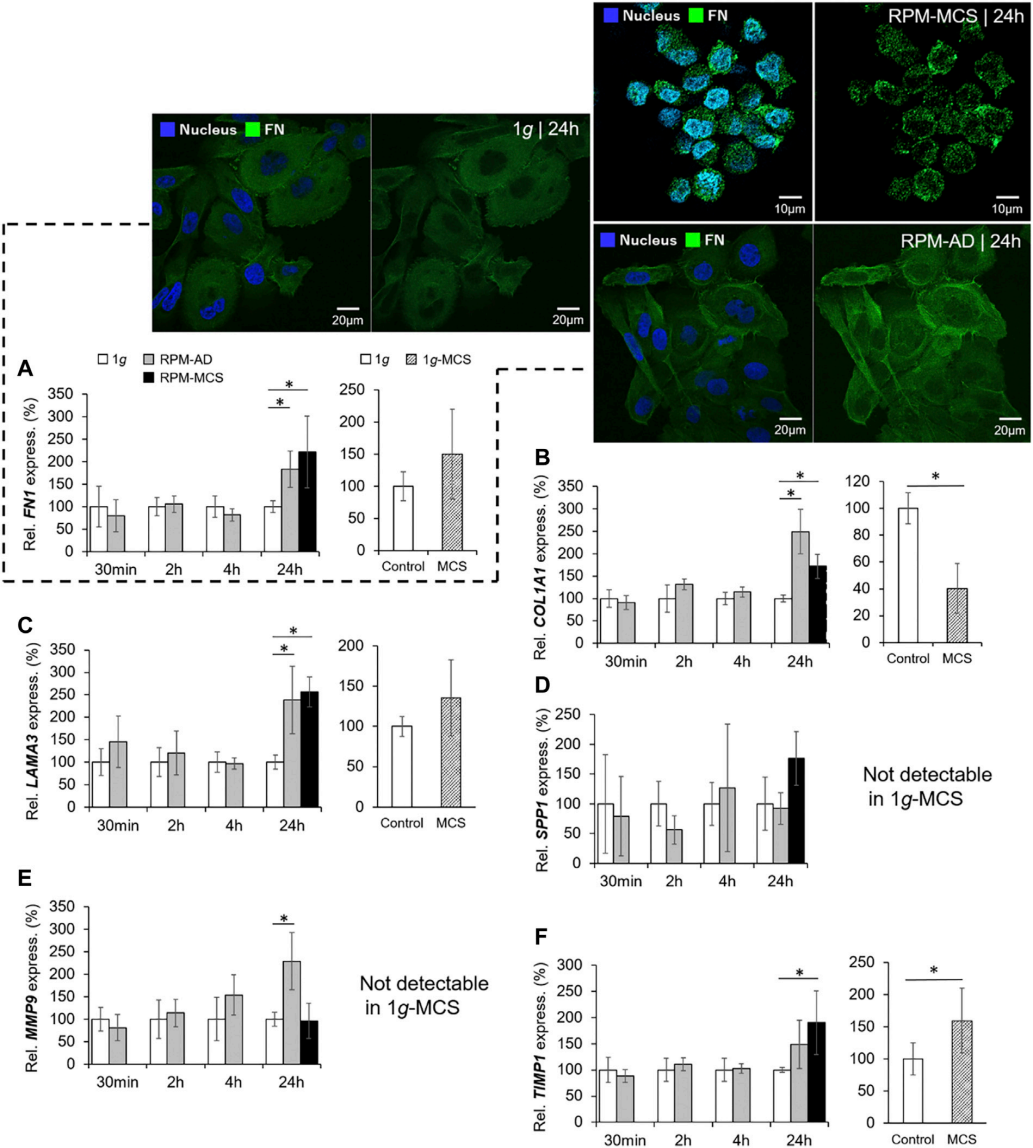
Extracellular matrix: Gene expression of **(A)** *FN1* and immunofluorescence of fibronectin: left two images of the 1*g*/24 h sample (merged left picture and fibronectin (Alexa Fluor 488) right picture), top right two images of the RPM/24 h MCS samples (merged left picture and fibronectin right picture) and lower right two images of the RPM/24 h AD samples (merged left picture and fibronectin right picture), DAPI-stained nucleus (blue), **(B)** *COL1A1*, **(C)** *LAMA3*, **(D)** *SPP1*, **(E)** *MMP9,* and **(F)** *TIMP1* of PC-3 cells exposed to the RPM for 30 min, 2, 4 and 24 h and 1*g*-MCS after 24 h. n = 5; *p < 0.05 vs. 1*g*.

The mRNA expression of *FN1* was significantly upregulated in PC-3 cells growing in the AD monolayer and in MCS when cultured under conditions of s-µ*g* for 24 h. Short-term s-µ*g* did not induce changes in the *FN1* gene expression of PC-3 cells. In addition, MCS grown under 1*g*-conditions revealed no expression changes of *FN1* (Figure 4A). The immunofluorescence staining revealed a similar amount of fibronectin in the cytoplasm of the PC-3 cells exposed for 24 h to the RPM compared to static control cells (Figure 4A, right image). MCS cells revealed a loose connection between neighboring cells and an uneven distribution of fibronectin within the cells which is in contrast to the 1*g* AD and RPM AD cells. In parallel, the PC-3 cells exposed to the RPM secreted a significantly reduced amount of fibronectin into the supernatant within 24 h (Table 3). The amount of secreted fibronectin was similar in all groups after 2 and 4 h of RPM exposure (Table 3).

**TABLE 3:** Secreted proteins of PC-3 cells [pg/mL]. n = 5; *p < 0.05 vs. 1*g*.

Protein	2 h		4 h		24 h	
	1*g*	RPM	1*g*	RPM	1*g*	RPM
IL-1α	22 ± 1	18 ± 2*	23 ± 2	25 ± 1	49 ± 8	42 ± 3
IL-1β	11 ± 1	10 ± 1	12 ± 2	15 ± 2*	22 ± 5	20 ± 2
IL-2	144 ± 2	132 ± 3*	146 ± 3	154 ± 3*	191 ± 13	184 ± 6
IL-6	13 ± 1	21 ± 3*	12 ± 3	58 ± 11*	59 ± 23	169 ± 42*
IL-7	14 ± 0	12 ± 0*	13 ± 1	15 ± 1*	21 ± 3	19 ± 2
IL-8	434 ± 65	195 ± 44*	519 ± 147	1,154 ± 208*	2,190 ± 1,059	3,097 ± 765
IL-17	39 ± 1	32 ± 4*	37 ± 5	40 ± 6	51 ± 7	48 ± 6
TNF-α	14 ± 1	11 ± 1*	14 ± 1	16 ± 1*	26 ± 4	23 ± 4
Fibronectin [ng/mL]	69 ± 2	58 ± 12	65 ± 13	65 ± 9	136 ± 31	97 ± 2*
Collagen I α1	125 ± 12	65 ± 11*	135 ± 33	138 ± 25	285 ± 101	163 ± 30*
Laminin	95 ± 9	145 ± 15*	88 ± 7	217 ± 34*	178 ± 17	233 ± 48
MMP-2	1854 ± 30	1786 ± 36*	1847 ± 39	1876 ± 45	2,149 ± 93	2068 ± 46
TIMP-1	1703 ± 208	1,334 ± 329	2,174 ± 695	1783 ± 372	9,245 ± 3,972	6,579 ± 1,086
Osteopontin	6,010 ± 154	5,441 ± 400*	5,928 ± 356	6,193 ± 260	7,287 ± 551	6,914 ± 537
EGF	13 ± 0	12 ± 2	12 ± 2	13 ± 2	15 ± 2	15 ± 2

Moreover, the *COL1A1* gene expression was not significantly changed when exposed to short-term (30 min, 2 h, 4 h) s-µ*g*. In contrast, after a 24 h RPM exposure there was a significant upregulation of *COL1A1* detectable in both AD and MCS samples while 1*g*-MCS revealed a significantly reduced expression of *COL1A1* (Figure 4B). In addition, collagen-1α1 was released by the PC-3 cells in a significant lower amount after a 2 and 24 h RPM exposure compared to control samples (Table 3).

A similar result in respect to the gene expression was found for *LAMA3* in 24 h RPM-exposed cells. *LAMA3* was significantly upregulated in AD and MCS after 24 h. No expression change was detected in 1*g*-MCS (Figure 4C). The secretion of laminin by the cells was significantly elevated after 2 and 4 h in RPM samples, but the release was not significantly altered in 24 h cultures (Table 3).

Furthermore, the *SPP1* mRNA was not changed when the cells were exposed to the RPM and was not detectable by qPCR in 1*g*-MCS (Figure 4D). The release of osteopontin into the cell supernatant was reduced early, but remained later unchanged compared to 1*g* samples (Table 3).

Finally, the gene expression of *MMP9* was significantly increased in AD cells after a 24 h-RPM-exposure and was not detectable in 1*g*-MCS (Figure 4E), whereas *TIMP1* was elevated in RPM-MCS as well as in 1*g*-MCS (Figure 4F). The secretion of TIMP1 was not changed at any timepoint (Table 1). After 2 h, the release of MMP-2 protein in the supernatant was significantly lower in RPM cultures but remained unchanged in 4 and 24 h cell cultures exposed to 1*g*- or RPM-conditions (Table 3).

### The Impact of Simulated Microgravity on Proinflammatory Cytokines

**FIGURE 5:**
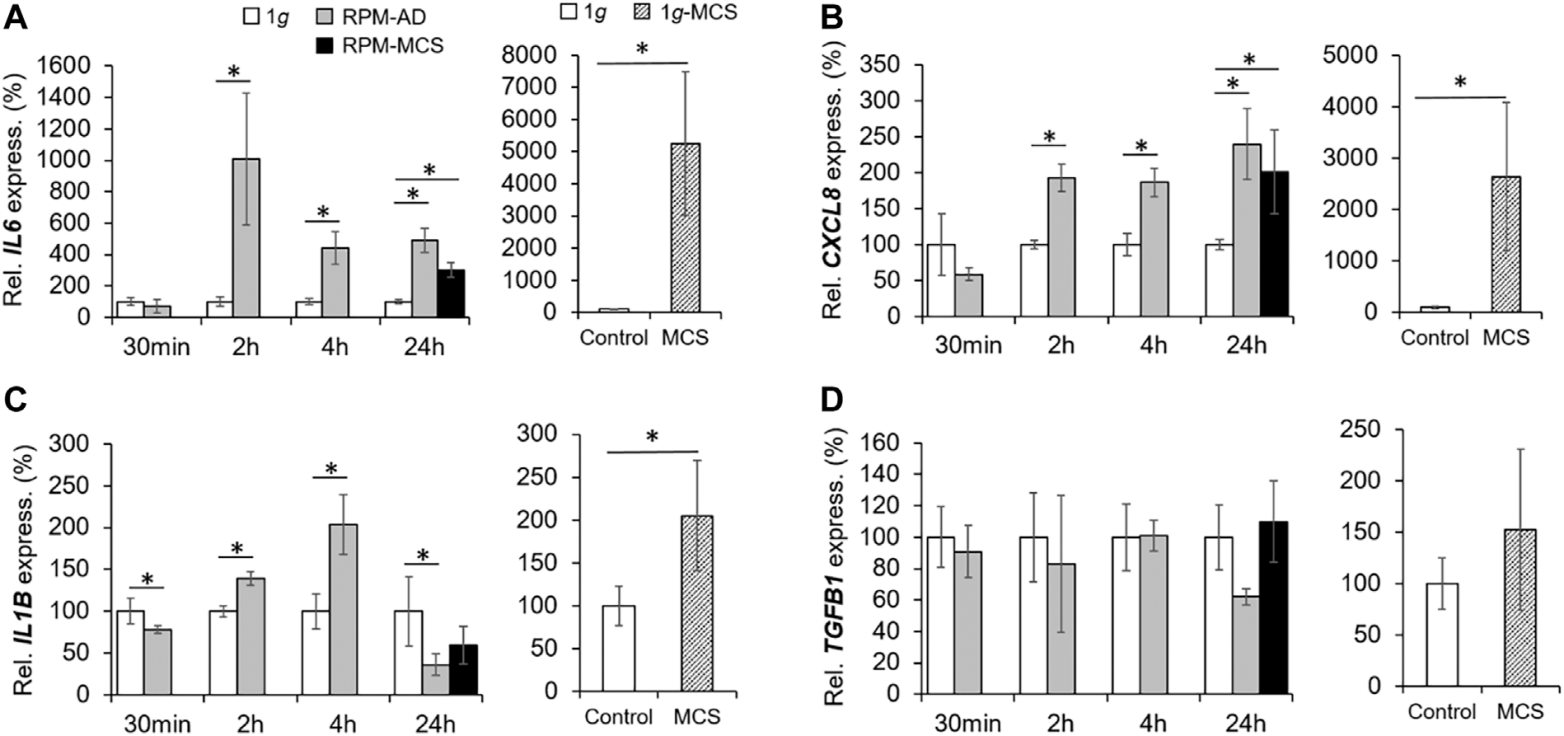
Proinflammatory cytokines: Gene expression of **(A)** *IL6* and **(B)** *CXCL8* mRNA, **(C)** *IL1B*, and **(D)** *TGFB1* of PC-3 cells exposed to the RPM for 30 min, 2, 4 and 24 h and 1*g*-MCS after 24 h. n = 5; *p < 0.05 vs. 1*g*.

We focused on the expression and secretion of proliferation of proinflammatory cytokines known to be involved in tumor progression and metastasis. A significant upregulation of *IL6* was found already after 2 h in RPM samples. The *IL6* gene expression remained elevated after 4 and 24 h in RPM samples (Figure 5A). The release of IL-6 in the supernatant was significantly enhanced in all RPM samples after 2, 4 and 24 h (Table 3).

In parallel, the *CXCL8* mRNA was significantly upregulated already after 2 and 4 h in PC-3 cells exposed to the RPM. In addition, *CXCL8* was elevated in AD after 24 h compared to 1*g* cells (Figure 5B). The amount of released IL-8 protein in the supernatant was reduced after 2 h, but clearly elevated after 4 h in RPM samples compared to corresponding 1*g* samples (Table 3). After 24 h the cells secreted an equal amount of IL-8 in the supernatant, irrespective of RPM exposure or static 1*g* culture conditions (Table 3).

The *IL1B* mRNA expression was downregulated in RPM samples after 30 min, then upregulated after 2 and 4 h and finally downregulated in AD and MCS samples after 24 h (Figure 5C). The IL-1β protein release in the cell supernatant by the PC-3 cells was not significantly altered in this time course (Table 3).

In contrast to these findings, *TGFB1* was not differentially expressed in 1*g* and RPM samples at all time points (Figure 5D). IL-17 protein was secreted in a significantly reduced amount in RPM-exposed PC-3 cells compared with 1*g* samples after 2 h (Table 3). In addition, TNF-α was also released in a significantly decreased amount by RPM-exposed PC-3 cells after 2 h but was elevated after 4 h (Table 3). The expression of proinflammatory cytokines in 1*g*-MCS was, with the exception of *TGFB1*, highly upregulated (Figures 5A–D).

### Impact of Simulated Microgravity on Anti-inflammatory Cytokines

**FIGURE 6:**
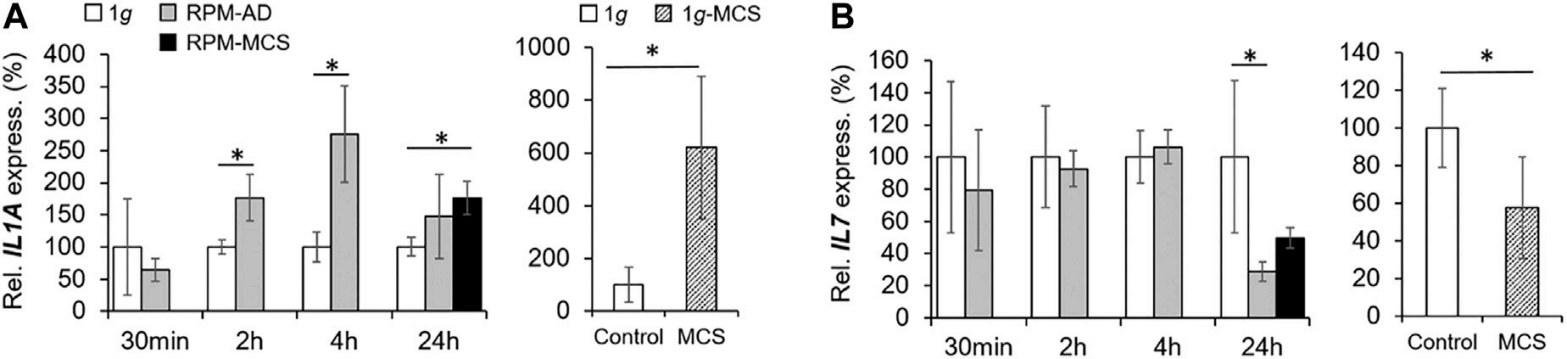
Anti-inflammatory cytokines: Gene expression of **(A)** *IL1A* and **(B)** I*L17* of PC-3 cells exposed to the RPM for 30 min, 2, 4 and 24 h and 1*g*-MCS after 24 h. n = 5; *p < 0.05 vs. 1*g*.

The gene expression of *IL1A* was elevated after 2 and 4 h in adherently growing cells exposed to the RPM compared to static control cells (Figure 6A). After 24 h, *IL1A* was significantly upregulated in MCS, but not in AD cells compared to 1*g* samples. The secretion of IL-1α in the cell supernatant was reduced in 2 h RPM-exposed samples compared to 1*g* (Table 3). In 1*g*-MCS a significant upregulation of *IL1A* was measured (Figure 6A).

Furthermore, we measured the release of IL-2 and detected a reduced amount of this protein after a 2 h RPM exposure of the cells, whereas after 4 h the secretion was elevated in the RPM samples (Table 3). In contrast, the *IL7* gene expression was not changed in short-term cultures (30 min, 2 and 4 h). Interestingly, in 24 h RPM cultures the *IL7* mRNA was significantly downregulated in AD and MCS cells compared to the control group, which is similar in 1*g*-MCS (Figure 6B). The secretion of the IL-7 protein was reduced after 2 h and enhanced after 4 h of RPM exposure of the cells (Table 3). After 24 h, the secretion of IL-7 was similar in both groups.

### Influence of Simulated Microgravity on VEGF, EGF and PI3K/AKT/mTOR (PAM) Signaling Pathways

The mRNA level of *VEGFA* was not changed in PC-3 cells exposed to the RPM for 30 min and 2 h. After 4 h the *VEGFA* mRNA was significantly downregulated in the AD group.

**FIGURE 7:**
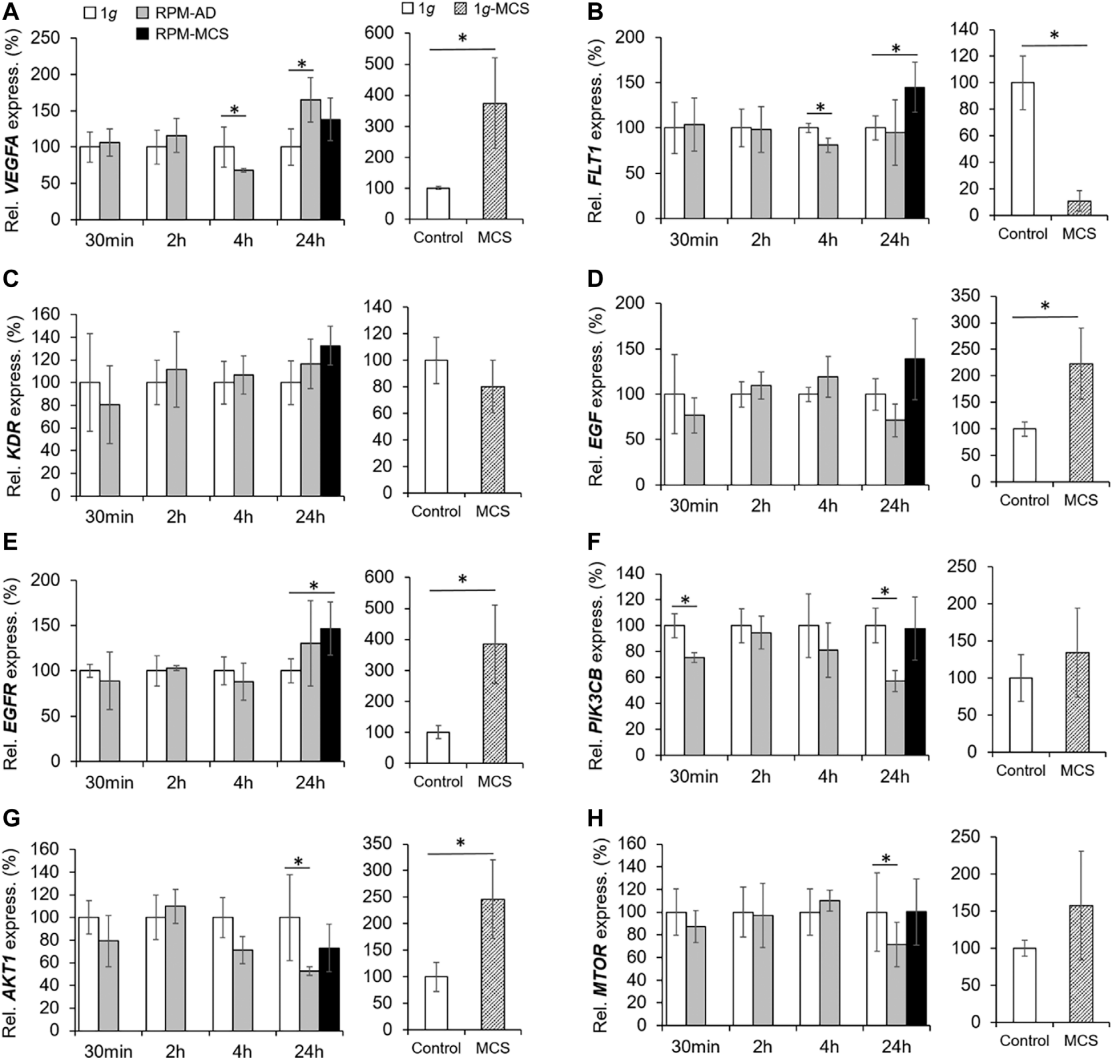
VEGF, EGF and PAM signaling: Gene expression of **(A)** *VEGFA*, **(B)** *FLT1*, **(C)** *KDR*, **(D)** *EGF*, **(E)** *EGFR*, **(F)** *PIK3CB*, **(G)** *AKT1,* and **(H)** *MTOR* of PC-3 cells exposed to the RPM for 30min, 2, 4 and 24 h and 1*g*-MCS after 24 h. n = 5 *p < 0.05 vs. 1*g*.

In contrast, the 24 h AD samples showed an increased level of *VEGFA* mRNA compared to 1*g.* RPM-MCS exhibited no change in *VEGFA*. In contrast, the gene expression of VEGF-A was elevated in 1*g*-MCS (Figure 7A). In parallel, we focused on the VEGF receptors *FLT1* and *KDR*. *FLT1* was significantly downregulated in AD cells after 4h, whereas after 24 h the *FLT1* mRNA was only upregulated in MCS. Contrary, *FLT1* is significantly downregulated in 1*g*-MCS (Figure 7B). The *KDR* mRNA expression was not altered over the entire time course using both methods (Figure 7C).

In addition, we investigated the *EGF* and *EGFR* gene expression in PC-3 cells exposed to s-µ*g* and in 1*g*-MCS. The *EGF* mRNA was not differentially displayed in PC-3 cells exposed to short-term microgravity. After 24 h, a non-significant increase in *EGF* was measured in MCS while a significant upregulation of *EGF* was measured in 1*g*-MCS (Figure 7D). After 24 h, a significant upregulation of the *EGFR* was detected in PC-3 cells growing in form of MCS using both methods (Figure 7E). The secretion of EGF protein was not changed in all groups (Table 3).

Furthermore, we studied key factors of the PAM pathway. The *PIK3CB* (Phosphatidylinositol-4,5-Bisphosphate 3-Kinase Catalytic Subunit Beta) gene was downregulated early in 30 min RPM exposed PC-3 cells (Figure 7F). After 24 h, RPM-exposed adherently growing PC-3 cells exhibited a downregulated *PIK3CB* mRNA expression. No expression changes were detectable in 1*g*-MCS (Figure 7F).

In parallel, the *AKT1* (RAC-alpha Serine/threonine-protein kinase 1) gene showed no altered expression in short-term samples but a significantly downregulated expression in 24 h AD RPM samples compared to 1*g.* Using the liquid overlay technique, the MCS group revealed a significantly upregulated *AKT1* (Figure 7G).

Finally, we studied the *MTOR* gene expression. The results were similar to the findings obtained for *AKT1. MTOR* was not differentially expressed in the short-term study, but the gene was downregulated in AD cells after a 24 h RPM exposure. In 1*g*-MCS s no expression changes were measured for *MTOR* (Figure 7H).

### Search Tool for the Retrieval of Interacting Genes/Proteins Analysis

**FIGURE 8:**
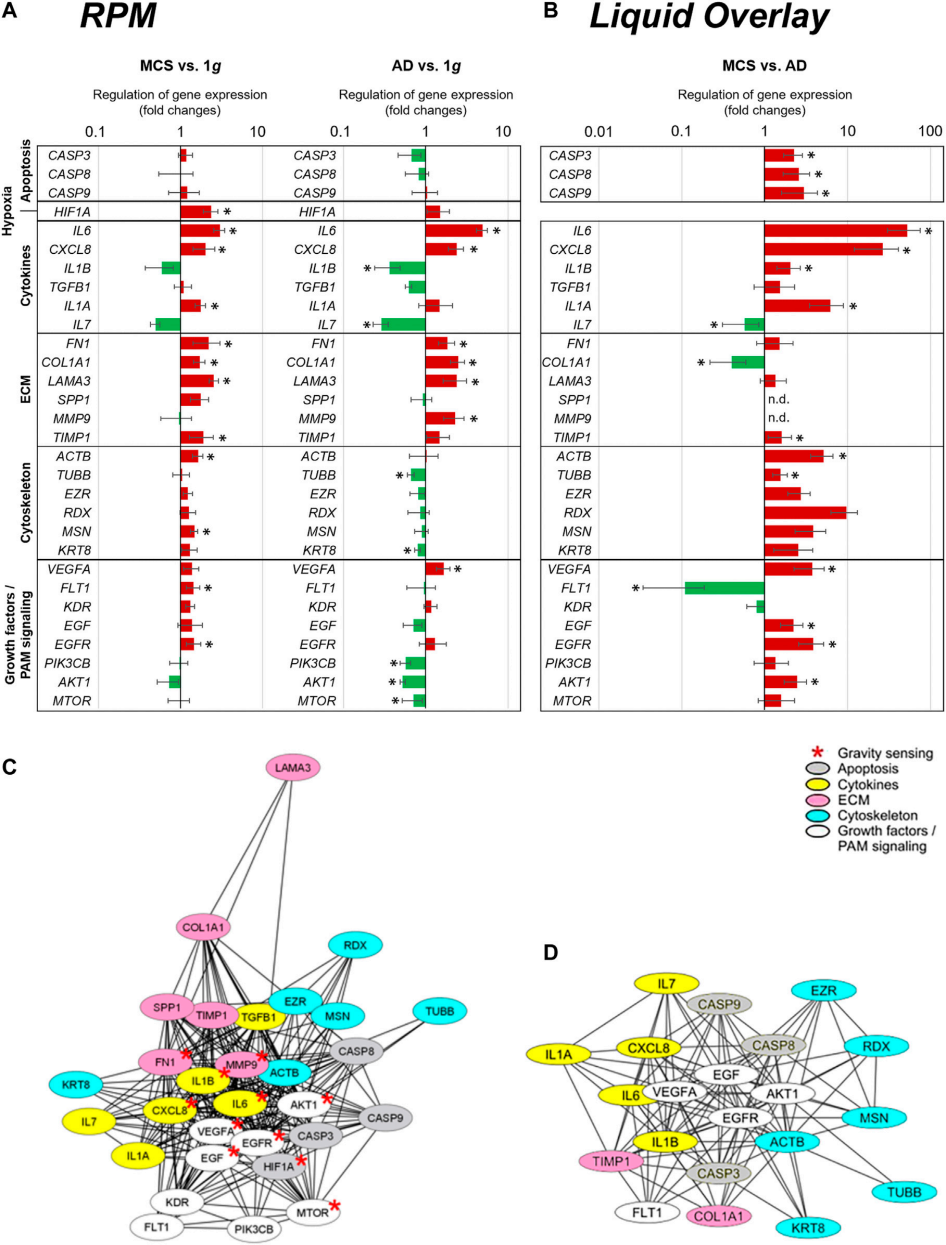
qPCR expression changes of selected genes and their relationship in STRING protein-protein interaction networks. **(A)** qPCR gene expression fold changes of the 24 h RPM-exposed PC-3 samples (AD and MCS) in relation to 1*g*. The red and green colors indicate upregulated and downregulated genes in RPM-exposed samples respectively. Significant regulations are indicated by black asterisks (*p* < 0.05). **(B)** qPCR gene expression fold changes of liquid overlay derived MCS cell aggregates in relation to controls. **(C,D)** Network of the functional interaction of genes and their products **(C)** analyzed in this study and **(D)** differtially regulated in liquid overlay derived MCS cell aggregates. The analysis was performed by STRING 11.0 (https://string-db.org/) and combined scores are visualized by Cytoscape 3.8.2. Affiliation to functional gene groups is color-coded. Red asterisks indicate genes known to be involved in prostate cancer gravity sensing.

Figure 8A presents a summary of the qPCR data, already demonstrated in Figures 1, 3–7, and gives an interpretation of the results. The genes of interest were differentially regulated in RPM samples (AD and RPM).

The results indicate several interactions for VEGFA, EGF, EGFR, IL1B, CXCL8, IL6, MTOR, AKT1, MMP9, and FN1, which are known to be involved in gravisensing of PC-3 prostate cancer cells exposed to short-term r-µ*g* (Figure 8C)*.* It became clear that these selected factors for which the expression pattern was measured are regulating each other very strongly.

The majority of the genes quantified in this study were upregulated in MCS with the exception of the downregulated *IL1B*, *IL7,* and *AKT1* mRNAs and *CASP3, CASP9, CASP8, MMP9, TUBB, EZR, RDX, KRT8, TGFB, SPP1, VEGFA, KDR, EGF, PIK3CB,* and *MTOR* which were not differentially displayed. A closer look at the 24 h AD samples revealed that the majority of cytoskeletal genes and PAM signaling factors were downregulated. In contrast, significant upregulations were found for ECM genes and proinflammatory cytokines such as among others *IL6*, *CXCL8*, and *VEGFA* and *FLT1*.

Figure 8B shows the summary of the qPCR data of the 1*g*-MCS vs. AD control cells (liquid-overlay engineered MCS), already given in Figures 2–7. The 1*g*-liquid overlay generated MCS cell aggregates show a different picture of gene regulation. Significant upregulations were measured for the following genes: *CASP3, CASP8, CASP9, IL6, CXCL8, IL1B, IL1A, TIMP1, ACTB, TUBB, EZR, RDX, MSN, KRT8, VEGFA, EGF, EGFR,* and *AKT1*. Significant downregulations were measured for *IL7, COL1A1,* and *FLT1*. In contrast to the RPM experiment, an upregulation of apoptosis-associated cysteine-aspartic acid proteases and a strong downregulation of the growth factor receptor FLT1 catch the eye (Figure 8B).

The various genes analyzed by qPCR were investigated with regard to possible interactions and mutual expression dependence of their corresponding proteins. A STRING/EMBL (European Molecular Biology Laboratory) analysis of the 30 qPCR items represented in this study are shown in Figure 8C. Figure 8D visualizes interactions of 21 proteins whose genes are significantly regulated in 1*g*-MCS compared to corresponding adherent controls. The STRING network shows a clear demarcation of cytokines from cytoskeletal genes.

## Discussion

For many years, it has been known that various cells exposed to r- and s-µ*g* exhibit a large number of morphological and molecular changes (Grimm et al., 2018; Grimm et al., 2020). Ingram et al. showed in 1997 that various tumor cell types, among them PC-3 cells, when exposed to s-µ*g* created by a NASA bioreactor grew in form of 3D aggregates (Ingram et al., 1997). The authors had used a NASA rotary cell culture system for the different spheroid cultures. They reported that the cell adhesion molecules CD44 and E-cadherin were upregulated in the 3D spheroids (Ingram et al., 1997). Furthermore, another group demonstrated the application of an s-µ*g* culture system to study growth and differentiation during a coculture of prostate stromal and epithelial cells on microcarrier beads (Zhau et al., 1997). The authors investigated the effects of an androgen (dihydrotestosterone) on growth and PSA expression by LNCaP prostate cancer cells grown alone or as coculture with prostate fibroblasts under s-µ*g* conditions (Zhau et al., 1997). The response was similar to that observed *in vivo*. Margolis et al. demonstrated that *ex vivo* integral prostatic tissue could be successfully cultured for 28 days on the NASA-designed Rotating Wall Vessel (RWV) (Margolis et al., 1999). The prostate tissue was still suitable for further investigations (Margolis et al., 1999). Another group used the high aspect rotating-wall vessel (HARV) to study the behavior of DU-145 human prostate carcinoma cells (Clejan et al., 2001). DU-145 cells exposed to HARV cultivation showed 3D growth as well as a less aggressive, slower growing, low proliferative, higher differentiated and less pliant cell than other techniques (Clejan et al., 2001).

The project Biotechnology Demonstration System-0, one of the 80 scientific experiments aboard Space Shuttle Columbia on flight STS-107, could show that in less than 1 day in space PCC had formed a tumor larger than one inch in diameter (Twombly, 2003). In a ground-based study under s-µ*g* conditions, the PCC and bone stroma formed small tissue aggregates (Twombly, 2003). On the space shuttle Columbia, the aggregates grew to the size of a golf ball by day 6. This data is in accordance with results obtained after the Shenzhou-8/SimBox Chinese-German space mission where follicular thyroid cancer cells (FTC-133 cell line) had been studied in space for 10 days (Pietsch et al., 2013). Interestingly, FTC-133 MCS grown in the flight sample in space ranged from 5 to 10 mm in diameter, while spheroids cultured on the RPM were significantly smaller at 2–3 mm in diameter, as observed in earlier experiments (Pietsch et al., 2010; Pietsch et al., 2013).

In a recent study, we investigated PC-3 cells for 3 and 5 days under s-µ*g* conditions using a desktop RPM without any scaffold (Hybel et al., 2020). The cells revealed changes in morphology, cytoskeleton, ECM, focal adhesion complex and growth behavior. In addition, a significant upregulation of genes belonging to the PAM pathway was demonstrated (Hybel et al., 2020).

### PC-3 Cells Exposed to the RPM Exhibit Changes in Morphology, Cytoskeleton and Extracellular Matrix

PC-3 cells cultured under conditions of s-µ*g* showed changes in growth within 24 h. One part grew in form of 3D multicellular spheroids and the other one continued growing as adherent cells in a 2D monolayer. This is in accordance with other cell types, including human thyroid cancer cells and breast cancer cells (Pietsch et al., 2010; Pietsch et al., 2013; Masiello et al., 2014). We focused on signs of apoptosis and could show that the TUNEL staining revealed no apoptotic cells after 24 h. In addition, the *CASP3, CASP8,* and *CASP9* genes were not differentially displayed. These findings demonstrated in Figure 1 show viable PC-3 after RPM exposure.

In addition, we have measured the gene expression of *HIF1A* after a 24-h exposure. Interestingly, there was no significant change in the adherently growing cells, but an increase in MCS (Figure 1H).

A significant *HIF1A* expression has been detected in a large number of cancers, which include among others prostate tumors (Zhong et al., 1999). Elevated HIF1A levels in several cancers have been associated with aggressive tumor progression, and thus has been implicated as a predictive and prognostic marker for resistance to therapy and increased mortality (Semenza, 2003). We do not expect hypoxia in the completely filled cell culture flasks because they have vented caps. The cells are viable and no apoptosis is detectable. The gas exchange has been studied earlier (Pietsch et al., 2012).

It is known that HIF1A is initiating angiogenesis through interactions with pro-angiogenic factors like VEGF-A (Birner et al., 2001). VEGF-A is elevated in RPM-exposed PC-3 cells after 24 h. HIF1A has a regulatory role in promoting tumor progression, likely through hypoxia-induced VEGF-A expression pathways (Powis and Kirkpatrick, 2004). HIF1A overexpression in tumors may also occur in a hypoxia-independent pathway. In hemangioblastoma, *HIF1A* expression is found in most cell samples from the well-vascularized tumor (Krieg et al., 2000). It will be of high interest to perform future long-term studies investigating this finding in detail.

A large number of studies have shown that r-µ*g* as well as s-µ*g* generated by an RPM has a major impact on the cytoskeleton (Vorselen et al., 2014; Corydon et al., 2016a; Chen et al., 2019; Nassef et al., 2019; Strube et al., 2020). Thereby it reacts on alterations of gravity with a bunch of rearrangements in the structure of the cytoskeleton. Moreover, it is suggested that the cytoskeleton may act as a direct sensor of gravity and displays the initial response to changed gravity levels (Vorselen et al., 2014; Häder et al., 2017). The cytoskeleton acts as a shape-giving structure providing the mechanical strength of cells. Thus, it works as a continuous pre-stressed lattice keeping cellular structural stability. It is composed of the actin and microtubule network, as well as intermediate filaments (Vorselen et al., 2014). F-actin belongs to the microfilaments and forms a large part of the cytoskeleton. In this study, we focused on the gene expression of β-actin (*ACTB*). β-actin is involved in forming the cell structure, cell motility and cell integrity. Short-term s-µ*g*-exposure induced no significant changes in the mRNA expression level (Figure 3). Remarkably, after 24 h an upregulation of *ACTB* was detectable in MCS samples but not in AD. This is in line with earlier studies finding that the mRNA level of ACTB was increased after cultivating PC-3 cells for 5 days on an RPM (Hybel et al., 2020). This supports the assumption that the actin-network is influenced by gravity changes and plays a critical role in 3D growth. Moreover, it is also suggested that actin itself can work as a mechanosensitive structure (Vorselen et al., 2014). The cytoskeleton of various cell types influenced by µ*g* reacts in a similar way, thus it can be assumed that the cytoskeleton acts as the general sensor of gravity and that the changes in the cytoskeleton become important for 3D growth (Grimm et al., 2018). Together with further morphogenetic events, these changes in the actin cytoskeleton promote the emergence of ordered structures and finally they result in the formation of MCS (Cui et al., 2017).

Moreover, we focused on the *TUBB* gene expression. β-tubulin belongs to the tubulin superfamily, that in turn contains six families (α, β, γ, δ, ε, and ζ tubulins). The most important families are the α- and β-tubulins as they form the major components of the microtubules. Short-term (30 min, 2 h, 4 h) RPM exposure showed no significant changes in the mRNA expression level compared to 1*g*. After a 24 h-exposure a significant downregulation was observed in AD samples but not in MCS samples. Previous research has clearly shown that microtubule self-organization is sensitive to the direction and the magnitude of gravity. The microtubules can respond to alteration of gravity by rearranging their structure and formation. Accordingly, they lose their radial organization, can be shortened, and can be more curved and bent (Lewis et al., 1998). But it was shown that these alterations are dependent on the type of cell and differ from cell to cell (Vorselen et al., 2014).

Furthermore, we investigated changes in the ECM. The ECM acts as a kind of sensor of alterations in gravity force. It is known that s-µ*g* exposure generated by an RPM, results in changes in the ECM like the formation of long-chain fibers with multiple RGD motifs. The RGD motif is a tripeptide that consists of arginine, glycine, and aspartate and mediates cell attachment. These motifs can bind tightly to the integrins on the cell membrane surface and interact with the cytoskeleton in the way that dispersed cells in the cell culture flask initially are drawn closer to form loose aggregates (Cui et al., 2017). In this manner the ECM is also early involved in spheroid formation of different tumor types (Grimm et al., 2002; Marrero et al., 2009; Lin et al., 2020).

To investigate the ECM components, we measured the *FN1*, *COL1A1*, *LAMA3, SPP1, MMP9* and *TIMP1* gene expression. Short-term s-µ*g* exposure (30 min, 2 h, 4 h) did not alter the expression of the selected ECM genes. In contrast, after a 24-h RPM-exposure, the *FN1*, *COL1A1* and *LAMA3* genes were elevated in both AD and MCS samples (Figures 4A–C). This is in line with earlier studies on different cell types demonstrating that there is a general tendency of elevated ECM components when cancer cells but also stem cells and specialized cells were exposed to long-term s-µ*g* (Grimm et al., 2002; Kraus et al., 2017; Ebnerasuly et al., 2018). It has to be noted that this increase is dependent on the cell type. There were opposite results detectable when adult retinal epithelium cells (ARPE-19) were exposed to the RPM (Corydon et al., 2016b). The *FN1* expression and *LAMB2* expression was reduced after RPM exposure in AD and MCS of ARPE-19 cells (Corydon et al., 2016b).

When analyzing the presence of fibronectin in MCS obtained in s-µ*g*, a slightly different cytoplasmic distribution, exemplified by a dotted pattern, compared to 1*g* samples was observed. A likely explanation could be that the cells of the MCS are significantly smaller. The apparent shrinking of the cells and the accompanying compression of the cytoplasm may collectively result in an altered cytoplasmic distribution and reduced secretion of fibronectin, despite the increased expression of *FN1*. However, reduced gravitational conditions may also impact the cytoplasmic appearance of fibronectin in RPM-MCS (Figure 4A).

In addition, human mesenchymal stem cells exposed to 10 days s-µ*g* showed a decrease in collagen production, as well as a reduced expression of *TIMP1*, *TIMP3,* and *MMP11* genes, together with an elevated expression of tenascin and laminin subunit (Zhivodernikov et al., 2020).

The *SPP1* gene expression was not significantly changed in all groups of PC-3 exposed to s-µ*g* conditions. The expression of osteopontin is known to be cell type-dependent. Rat osteoblasts cultured for 4 or 5 days aboard the Space Shuttle revealed a reduced (30%) *SPP1* mRNA (osteopontin) in the flight samples (Kumei et al., 2006). In contrast, the *SPP1* mRNA was elevated in human fetal osteoblasts exposed to the RPM (Mann et al., 2019). A similar result was obtained for human primary chondrocytes (Wehland et al., 2020).

### Simulated Microgravity Influences the Expression and Release of Inflammatory Cytokines in PC-3 Cells

We focused on the cytokine release pattern of PC-3 cells when exposed to the RPM. It is assumed that some cytokines play a major role in spheroid formation. *IL6* for example exhibits a higher expression in PC-3 cells as well as in other prostate cancer cell lines and plays a major role as a proliferative autocrine and paracrine factor in prostate cancer (Azevedo et al., 2011). Furthermore, Gopinathan et al. (Gopinathan et al., 2015) showed that *IL6* can directly generate the development of new blood vessels, the proliferation and migration of endothelial cells and has thereby a tumor promoting activity.

In the present study, the *IL6* gene expression increased very early with a 10-fold peak after 2 h of RPM-exposure. Afterwards, within 4 and 24 h (AD and MCS) it slightly decreased but still showed an about 5-fold elevation compared to 1*g* samples. This is in line with Grosse et al. (Grosse et al., 2012), who performed similar experiments with FTC-133 thyroid cancer cells and demonstrated that the tumor cells on the RPM released IL-6 in the supernatant. Svejgaard et al. (Svejgaard et al., 2015) demonstrated that both cytokines IL-6 and IL-8 improve 3D aggregation of the human thyroid cancer cell lines (ML-1 and RO-82-W-1) using the liquid overlay technique and that these cytokines induced the protein expression of β-actin, β_1_-integrin, talin-1, and Ki-67. These findings implicate that IL-6 as well as IL-8 are involved in spheroid formation. The detailed mechanisms are still unknown and have to be investigated more precisely in future studies. Interestingly, the IL-6 release was significantly elevated in all RPM samples at all time points (Table 3).

Taken together, all these findings indicate that interleukin-6 might be an important factor for tumor cell growth, angiogenesis, metastasis and spheroid formation. The expression of *CXCL8* showed a similar pattern as *IL6* indicating a similar reaction of the anti-inflammatory cytokines to microgravity.

Singh et al. (Singh and Lokeshwar, 2009) showed that IL-8 is acting as a survival factor of cancer cells and in this context IL-8 interacts with Akt and NF-κB, and has thereby a control function on the apoptotic pathway. Moreover, IL-8 plays a role in PC-3 survival, invasion, and resistance to chemotherapeutic drugs in PC-3 cells. Wilson et al. (Waugh and Wilson, 2008) mentioned that IL-8 signaling is involved in PC-3 survival and acts as an intrinsic factor of chemoresistance in advanced prostate cancer. Besides, Waugh et al. (Waugh and Wilson, 2008) showed that *CXCL8* signaling regulates, among others, the transcriptional activity of the androgen receptor of PC-3 so that PC-3 proliferate androgen-independently. Therefore, taken these findings together, IL-8 is of special interest as it has an impact on PC-3 cells in many ways.

In the present study, we measured an upregulation of *CXCL8* in AD cells and 3D PC-3 MCS. The secretion of IL-8 was significantly elevated after 4 h, but similar after 24 h between the RPM and 1*g* group (Table 3). It is known that IL-8 increases the expression of several proteins of the cytoskeleton and focal adhesion complex. These proteins in turn play a major role in tumor progression and metastasis (Desiniotis and Kyprianou, 2011). Remarkably, these proteins belonging to the cytoskeleton and focal adhesion complex also can sense gravity changes and therefore have a great impact on spheroid formation.

### Simulated Microgravity has Impact on VEGF, EGF, and PAM Signaling

We studied factors of signaling pathways known to be involved in 3D growth (Krüger et al., 2019; Nassef et al., 2020). The expression of genes belonging to the *VEGF* signaling pathway were analyzed, showing that the gene expression of *VEGFA* was downregulated after 4 h and in contrast upregulated after 24 h of RPM exposure in AD samples compared to 1*g* (Figure 7A). In MCS samples, however, the *VEGFA* mRNA showed no significant change. One potential explanation for this finding could be that a less-aggressive phenotype developed when the cells merged into spheroid formation, which was found earlier in follicular thyroid cancer cells cultured in space (Ma et al., 2014). Furthermore, VEGF-A has various well-known effects in cancer. It is mediating increased vascular permeability, inducing angiogenesis, vasculogenesis and growth. In addition, VEGF-A promotes migration and progression. Within 24 h, the prostate cancer cells exposed to the RPM start to detach and to form MCS. A high amount of VEGF-A promotes spheroid formation which might explain the elevated level of *VEGFA* mRNA in 24 h AD samples.

The *KDR* gene was not differentially expressed in all groups, whereas *FLT1* was downregulated after 4 h in AD, unchanged after 24 h in AD, but elevated in MCS. As the VEGF-A pathway has been implicated in pathological angiogenesis and tumor development (Nagy et al., 2007), a lower expression of the pathway points towards a less-aggressive cancer growth behavior.

Furthermore, we investigated the gene expression of *EGF*. The EGF protein is a key player in cancer by enhancing cell proliferation, survival, invasion, and metastasis (Bhat et al., 2014). The expression of epidermal growth factor receptor (EGFR) in cancer is often associated with a more aggressive phenotype and predictive of poor prognosis (Bhat et al., 2014). The *EGFR* mRNA is upregulated in MCS compared to 1*g* control cells indicating its involvement in 3D spheroid formation (Figure 7E).

The PAM pathway is of special interest because it is often mutated in prostate cancer (Tee et al., 2018) and therefore involved in cancer growth and progression. It is also a frequent reason of drug-resistance especially to androgen-deprivation therapy in prostate cancer (Park et al., 2018). Regarding the PAM signaling pathway, the *PIK3CB* gene was downregulated after 30 min and finally, after 24 h also in AD samples. A similar result was obtained for the *AKT1* and *MTOR* mRNAs, which were both reduced in 24 h AD cells. In addition, these genes were not altered in MCS. This is an interesting result because downregulation of the PAM pathway can activate apoptosis in cancer (Yang et al., 2019). Apoptosis was not detected after RPM exposure, which is an interesting result and it can therefore be concluded that other signaling factors exhibited anti-apoptotic effects on the PC exposed to short-term µ*g*. Long-term s-µ*g*-exposure of PC-3 cells (5 days) resulted in a significant upregulation of *AKT* and *MTOR* mRNAs in both AD and MCS (Hybel et al., 2020).

### Interaction Network of Selected Genes Evaluated by STRING Analysis and Cytoscape 3.8.2

The STRING analysis revealed an interaction network of VEGFA, FLT1, EGF, EGFR, IL1B, IL6, CXCL8, MTOR, AKT1, MMP9, and FN1*.* The interaction between the VEGF and EGFR pathway is well known and the rationale for a multi-target anticancer therapy (Ciardiello et al., 2006). EGF application is able to enhance VEGF-A production and to induce PI3K-dependent positive feedback on AKT and ERK via VEGFR2 in hematological malignancies (human monocytic leukemia THP1 cell line and Burkitt’s lymphoma Raji cell line (Saryeddine et al., 2016). Both pathways are key players in cancer cell growth, progression, metastasis and angiogenesis. Multikinase inhibitor therapy targeting among other factors VEGF-A is applied today in different types of advanced metastatic cancers (Wehland et al., 2012; Ancker et al., 2017; Randrup Hansen et al., 2017; Sarkar et al., 2020). EGF mediates cellular proliferation, differentiation, and survival (Herbst, 2004) and is involved in spheroid formation of cancer cells exposed to r-µ*g* in space. The *EGF* gene expression was clearly upregulated in AD and MCS of FTC-133 follicular thyroid cancer cells in space during the Shenzhou-8 space mission (Pietsch et al., 2013). These results indicate the importance of EGF signaling for spheroid formation.

A further factor involved in spheroid formation and spreading of cancer cells is fibronectin. Proteome analyses revealed that surface proteins are binding fibronectin, and thus strengthening the 3D spheroid formation of thyroid cancer cells (Pietsch et al., 2011). This might be also important for other cancer types. Bioinformatic analyses have demonstrated that EGFR, KDR, FN1, TGFB1 as well as PCNA are interacting with VEGF-A and are involved in non-small cell lung cancer tumorigenesis (Wang et al., 2015). FN1 is involved in the occurrence and development of various tumors and is upregulated in multiple cancer types. *FN1* is able to promote cell proliferation and migration in gastric cancer cell lines (Sun et al., 2020). A recent study showed that both cell adhesion molecules and ECM components OPN (SPP1) and FN1 might work as biological markers of progression and prognosis in esophageal cancer (Li et al., 2020).

The functional roles of VEGF and OPN in angiogenesis and their clinical significance in tumor biology are well-described (Shijubo et al., 2000). In PC metastasis both protein synthesis and gene expression of *SPP1* were remarkably upregulated in metastatic castration-resistant PC (Pang et al., 2019).

Pro-inflammatory cytokines such as IL-1, IL-6, IL-17, and TNF-α promote proliferation and differentiation of cancer cells (Vendramini-Costa and Carvalho, 2012). The cytokines IL-6 and IL-8 (CXCL-8) are further key elements which are able to enhance 3D growth in PC-3 and have both already shown to induce 3D growth in thyroid tumor cells grown under 1*g*-conditions using the liquid overlay technique (Svejgaard et al., 2015). Both cytokines were clearly elevated in PC-3 exposed to the RPM and may serve as key players for 3D aggregation. IL-6 is a key factor in the tumor microenvironment. IL-6 overexpression was demonstrated in almost all cancer types (Kumari et al., 2016). High levels of IL-6 advance tumorigenesis and regulate among others metabolism, angiogenesis, invasiveness, metastasis, apoptosis, and survival (Kumari et al., 2016). IL-6 can induce cell growth and VEGF synthesis in malignant mesotheliomas or gastric cancer (Huang et al., 2004; Adachi et al., 2006). Furthermore, EGFR signaling promotes induction of the IL-6 receptor controlled by mTOR (Garbers et al., 2013). An aberrant EGFR activation triggered IL-6 synthesis (Garbers et al., 2013).

The PI3K-AKT-mTOR signaling network is activated and during prostate tumorigenesis, PC progression and recurrence (Shorning et al., 2020). The mTOR pathway is involved in VEGF biosynthesis, and disruption of the VEGF/Neuroplin-1 (NRP1) axis. VEGF/NRP1 are promoting angiogenesis and pro-tumorigenic signaling in both endothelial and cancer cells (Pal et al., 2019). The *VEGFA* gene expression is enhanced in AD cells indicating signaling towards 3D formation of PC-3.

Even though there are various more convenient techniques to produce spheroids like the hanging drop technique (Timmins et al., 2004) or the liquid-overlay technique (Svejgaard et al., 2015), these methods introduce unfavorable aspects which are low quantity, poor nutrition exchange among others. In addition, the transition from 2D growth to 3D growth cannot be monitored in these experimental setups. We used the liquid-overlay technique and engineered MCS under 1*g*-conditions. After 24 h, the PC-3 cells formed loose 3D aggregates on agarose. Unfortunately, more dead cells were detected compared to RPM-engineered MCS (Figure 2B). The qPCR analysis revealed a strong up-regulation for proinflammatory cytokines like among others *IL6* and *CXCL8.* This finding might be explained by the higher amount of dead cells in 1*g*-engineered MCS. The opposite result was obtained for the expression of *VEGFA* and *FLT1* in 1*g*-MCS. *VEGFA* and *KDR* were both not differentially altered and *FLT1* was significantly elevated in RPM-MCS, whereas *VEGFA* was upregulated and *FLT1* down-regulated in 1*g*-MCS.

In addition, the ECM genes *FN1, COL1A1,* and *LAMA3* were all significantly up-regulated in RPM-MCS and differentially regulated in 1*g*-MCS. Taken together the results involving 1*g*-MCS engineered with the liquid-overlay technique are not suitable to study the early phases of tumor progression and metastasis in PC. The MCS formed with an RPM are rounder and compact, are created without agarose or a scaffold, do not show an increase in apoptosis and can grow for a longer time under s-µ*g* conditions as shown in an earlier study (Hybel et al., 2020).

### Comparison Between Short-Term and Long-Term Changes in PC-3 Cells Exposed to the RPM

*COL1A1,* which encodes one part of the fibril-forming pro-alpha1 chains of type I collagen, *LAMA3,* encoding the alpha part of the heterotrimeric laminin molecule, and *FN1,* encoding fibronectin, which is involved in RET signaling and is part of the integrin pathway, are substantial components of the ECM. All three genes are upregulated in PC-3 after a 24-h RPM-exposure in AD as well as in MCS. In a previous study with long-term RPM-exposure of PC-3 cells, we found that the upregulation of *LAMA3* and *FN1* expression persists after 3 days of RPM exposure in AD and MCS but turns to depletion in AD at day five (Hybel et al., 2020). In contrast, the *COL1A1* gene is up-regulated after 5 days of RPM-exposure (Hybel et al., 2020).

*ACTB* and *TUBB* are encoding the β-actin and β-tubulin class I proteins, respectively. Both factors are substantial proteins of the cytoskeleton. While the *ACTB* expression is upregulated in PC-3 cells after a 24-h RPM-exposure only in MCS, the *TUBB* expression is depleted after 24 h in RPM-AD samples. However, after 5 days under RPM-conditions, both genes are upregulated in AD as well as in MCS samples (Hybel et al., 2020).

*AKT1* encodes one of the three AKT serine-threonine protein kinases and participates in mTOR signaling. Both, *AKT1* and *MTOR* are depleted in AD under 24 h in s-µ*g,* but upregulated after 5 days in s-µ*g* (AD & MCS) (Hybel et al., 2020).

In contrast, *FLT1* and *VEGFA,* encoding the angiogenesis proteins vascular endothelial growth factor receptor 1 and the vascular endothelial growth factor 1, are both upregulated after 24 h s-µ*g*. The up-regulation of *FLT1* takes place in MCS and the up-regulation of *VEGFA* in AD. After 5 days of µg exposure *FLT1* is upregulated in AD and MCS and *VEGFA* expression is depleted in MCS (Hybel et al., 2020).

In general, compared to a short-term s-µ*g-*experiment, substantial changes in AD and MCS expression of cytoskeletal genes, extracellular matrix and PAM signaling can still be detected after three and 5 days of RPM-exposure, respectively. This suggests that in the future extended time course experiments may be appropriate. Recent studies have highlighted the existence of an integrated signaling network connecting mechanosensitive pathways to circadian gene regulation in *e.g.* human keratinocytes (Ranieri et al., 2015). Whether this is also the case in PC-3 prostate cancer cells awaits further studies.

In summary, this study focused on the early effects of s-µ*g* on PC-3 cells. Short-term s-µ*g* influenced the growth behavior of PC-3 cells towards a 3D phenotype. No signs of apoptosis were detectable. Changes in the expression of genes belonging to the cytoskeleton, ECM, cytokines, VEGF, EGFR, and PAM signaling were measured. This was accompanied by alterations of the secretion of the cytokines and ECM components. We observed significant increases in *IL6* and *CXCL8* gene expression after 2, 4 and 24 h in MCS, which hints towards a more aggressive phenotype in short-term microgravity. After 24 h *TIMP1* was elevated in MCS and *MMP9* in AD cells (Figures 4E,F). In addition, the release of IL-6 in the supernatant was elevated at all time points in RPM samples. These results fit to earlier short-term studies (parabolic flight experiments) which have already shown that thyroid cancer cells exhibit a more aggressive phenotype when cultured under r-µ*g* (Ma et al., 2014). This is a finding which should be studied in more detail in the future.

PC-3 exposed to s-µ*g* created by an RPM grew in form of two phenotypes: an adherent monolayer and as 3D aggregates. The PC-3 cells started to aggregate 24 h following subjection to s-µ*g* conditions. Moreover, the 24 h RPM exposure of PC-3 cells resulted in an early activation of the VEGF pathway, EGFR1 and a downregulation of PAM signaling. Moreover, the secretion and gene expression of proinflammatory cytokines *IL1B*, *IL6* and *CXCL8* were markedly upregulated and closely involved in the first phases of spheroid formation of PC-3 cultivated under conditions of s-µ*g*. This makes them interesting targets for a possible suppression of the development of metastases. In fact, HuMax-IL-8 (BMS-986253), a novel fully human monoclonal anti-IL-8 antibody has recently been introduced in different phase I trials testing its anti-cancer potential [NCT02536469, NCT03689699] (Bilusic et al., 2019). These trials, however, were done on patients with advanced, already metastasized stages of cancer. Our results suggest that IL-6 or IL-8 inhibition might already be beneficial in early stages of cancer by preventing or slowing down metastasis. Both factors will be targeted in future short- and long-term experiments. Liquid-overlay engineered PC-3 MCS revealed apoptotic cells after 24 h, which may influence the expression of cytokines, cytoskeletal genes and other factors. Taken these findings together, multicellular spheroids engineered by microgravity represent a novel model for studying the early phases of metastasis *in vitro*. The present findings may thus provide additional insights in selecting new targets to impair prostate cancer progression.

## Data Availability

The raw data supporting the conclusion of this article will be made available by the authors, without undue reservation.

## Glossary

µ*g*: Microgravity
2D: Two-dimensional
3D: Three-dimensional
ACTB: Beta-actin
AD: Adherent Monolayer
AKT1: RAC-alpha Serine/Threonine-protein kinase 1
CASP3: Caspase-3
CASP8: Caspase-8
CASP9: Caspase-9
COL1A1: Collagen-1a1
CXCL8/IL-8: Interleukin-8
ECM: Extracellular matrix
EGF: Epidermal Growth Factor
EGFR1: Epidermal Growth Factor Receptor 1
EZR: Ezrin
FLT1: Tyrosine kinase 1
FN1: Fibronectin
HARV: High aspect rotating-wall vessel
HIF1A: Hypoxia inducible factor 1A
IL-17: Interleukin-17
IL-1A: Interleukin-1 Alpha
IL1B: Interleukin-1 Beta
IL-2: Interleukin-2
IL-6: Interleukin-6
IL-7: Interleukin-7
KDR: Kinase Insert Domain Receptor
KRT8: Kreatin 8
LAMA3: Laminin Subunit Alpha 3
LNCaP: Lymph Node Carcinoma of the Prostate
MCP-1: Monocyte Chemoattractant Protein 1
MCS: Multicellular Spheroid(s)
MMP11: Matrix Metalloproteinase-11
MMP2: Matrix Metallopeptidase-2
MMP9: Matrix Metalloproteinase-9
MSN: Moesin
MTOR: Mechanistic Target of Rapamycin kinase
NFKB: Nuclear Factor kappa B
NRP1: Neuroplin-1
OPN/SPPI: Osteopontin/Secreted Phosphoprotein 1
PAI-1: Plasminogen Activator Inhibitor-1
PCC: Prostate Cancer Cells
PCNA: Proliferating Cell Nuclear Antigen
PFA: Paraformaldehyde
PIK3CB: Phosphatidylinositol-4,5-bisphosphate 3-kinase Catalytic Subunit Beta
r-µ*g*: Real Microgravity
RDX: Radixin
RPM: Random Positioning Machine
RWV: Rotating Wall Vessel
s-µ*g*: Simulated Microgravity
TGFB1: Transforming Growth Factor Beta 1
TIMP1: Tissue Inhibitor of Metalloproteinases/metallopeptidase Inhibitor 1
TIMP3: TIMP metallopeptidase Inhibitor 3
TNF-a: Tumor Necrosis Factor Alpha
TUBB: Tubulin Beta
VEGFA: Vascular Endothelial Growth Factor A
